# The Effect of Bacterial Infections, Probiotics and Zonulin on Intestinal Barrier Integrity

**DOI:** 10.3390/ijms222111359

**Published:** 2021-10-21

**Authors:** Paweł Serek, Monika Oleksy-Wawrzyniak

**Affiliations:** 1 Department of Biochemistry and Immunochemistry, Wroclaw Medical University, 50-368 Wroclaw, Poland; 2 Department of Pharmaceutical Microbiology and Parasitology, Wroclaw Medical University, 50-556 Wroclaw, Poland; em.oleksy@gmail.com

**Keywords:** intestine barrier, gut microbiota, zonulin, probiotics, bacteria, infection

## Abstract

The intestinal barrier plays an extremely important role in maintaining the immune homeostasis of the gut and the entire body. It is made up of an intricate system of cells, mucus and intestinal microbiota. A complex system of proteins allows the selective permeability of elements that are safe and necessary for the proper nutrition of the body. Disturbances in the tightness of this barrier result in the penetration of toxins and other harmful antigens into the system. Such events lead to various digestive tract dysfunctions, systemic infections, food intolerances and autoimmune diseases. Pathogenic and probiotic bacteria, and the compounds they secrete, undoubtedly affect the properties of the intestinal barrier. The discovery of zonulin, a protein with tight junction regulatory activity in the epithelia, sheds new light on the understanding of the role of the gut barrier in promoting health, as well as the formation of diseases. Coincidentally, there is an increasing number of reports on treatment methods that target gut microbiota, which suggests that the prevention of gut-barrier defects may be a viable approach for improving the condition of COVID-19 patients. Various bacteria–intestinal barrier interactions are the subject of this review, aiming to show the current state of knowledge on this topic and its potential therapeutic applications.

## 1. Introduction

The main responsibility of the digestive system is digesting food and absorbing the highest possible content of nutrients obtainable from consumed products. The role of the digestive system (especially the intestine) is predominantly limited solely to these functions. In fact, the intestine can be defined as the pathway that connects the external and internal environments of the body, and its serves as a determinant for what components enter the bloodstream and in what quantity. Therefore, the intestinal barrier plays a vital role in maintaining immune homeostasis of the gut and the entire body. An observation has been made that a shift in the intestinal microbiota composition balance towards opportunistic microorganisms results in the increased secretion of a recently discovered protein, zonulin. Studies have documented that increased zonulin expression and intestinal barrier permeability are interconnected with celiac disease, type 1 diabetes, and other autoimmune diseases. This indicates that recent advances on the topic of intestinal microbiota and its relationship with the physiology of intestinal diseases necessitate this review and update of the contemporary literature on this topic.

## 2. Intestinal Barrier

There is a large body of literature available on the topic of the intestinal barrier, its permeability and importance for homeostasis, and it clearly indicates that these are very complex issues [1,2,3,4,5]. The intestinal barrier is the largest surface in the human body that is in contact with the external environment, which enables it to possess the ability to dynamically respond to its factors. It is the site of interaction with orally delivered stimuli, while simultaneously constituting a barrier against penetration by pathogens, toxins and antigens. Food is digested in the intestinal lumen—an environment formed by bile, pancreatic juice and enzymes—where antigens and microorganisms are also degraded [6].

**Figure 1:**
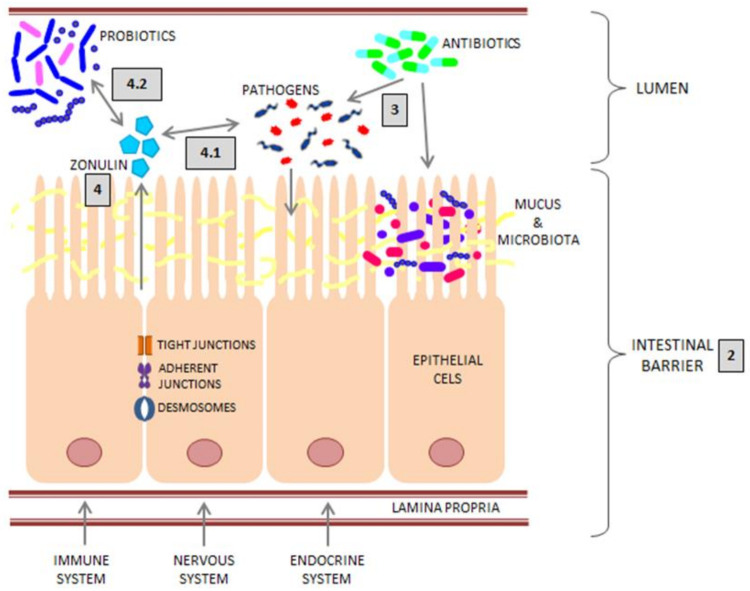
Model of the intestinal barrier and the scheme of its interactions with pathogenic bacteria, antibiotics, probiotics and zonulin. The structure of the intestinal barrier (reviewed in Section 2). Antibiotics and pathogenic bacteria (reviewed in Section 3). Zonulin and its interaction with the barrier and the influence on its secretory function (reviewed in Section 4).

Its structure is subject to dynamic changes and is determined by physical, chemical and biological components such as mucus, cells (epithelial, secretory and immune) and bacterial microflora [7]. All the components of the intestinal barrier, as well as its interaction with bacteria and zonulin, are shown in Figure 1.

### 2.1. Epithelium

The small intestine epithelium consists of a single layer of cylindrical enterocytes, goblet cells, Paneth cells, enteroendocrine cells, microfold cells (M cells), cup cells and tuft cells, although the functions of the latter two are still not fully understood [8]. To provide the lowest level of permeability to antigens while allowing the influx of ions and solutes, adjacent epithelial cells are connected by an “apical junctional complex” consisting of tight junctions (zonula occludens) (TJs), adherens junctions (zonula adherens) (AJ) and desmosomes [9]. TJs are composed of three major transmembrane proteins: occludin, claudins and junctional adhesion molecules (JAMs). These bind to various peripheral membrane proteins, such as ZO-1 (Zonula occludens-1, also known as tight junction protein-1) located on the inside of the cell membrane, anchoring the actin components of the cytoskeleton. AJ is formed by E-cadherin, α-catenin and β-catenin [7]. TJs are involved in cell polarity and signaling by regulating ion and molecule transport across the epithelium, making them an essential component to maintain intestinal homeostasis [4]. Intercellular junction proteins can exhibit different properties. For example, claudins 1, 3, 4, 5 and 8 are used to reinforce the barrier, while claudins 2, 7, 10 and 23 tend to weaken it and increase permeability [1,5]. The epithelial cells are based on a connective tissue thin membrane–lamina propria. This structure enables the innate and acquired mechanisms of the immune system (class A immunoglobulins, cytokines, proteases and chemokines) to function, as well facilitates functioning of the endocrine and nervous systems that control intestinal motility [6]. In terms of ultrastructure and function, the cell barrier shows considerable regional variation along the intestine, with the colonic barrier being less permeable than the small intestine. Differences in small intestine permeability and pore size are also observed locally, varying from 4–5 Å at the ends of the villi to more than 20 Å at the base of the crypt [3].

### 2.2. Transport across the Intestinal Barrier

There are two types of transport pathways through the epithelium: paracellular (between neighboring cells, caused by dynamic opening and closing of intercellular junctions), and transcellular (through endothelial cells by endocytosis). The cBasic ultrastructure and biology of the tight junctions of paracellular pathways, which hold the critical role of fluid and electrolyte absorption, have been of great interest [1,2,5]. The paracellular permeation of molecules is mainly controlled by TJs, which regulate the influx of ions and other small molecules of molecular weight less than 600 Da through the intestinal wall [10]. The tight junction barrier exhibits selectivity in transporting molecules and enforces this feature by assessing both their size and charge. There are two variants of transport across junctions with an intact epithelial monolayer, called the “pore” and “leak” pathways. A pore path corresponds to a high-capacity, size- and charge-selective path, while a leak pathway is a low-capacity pathway with limited selectivity. The pore pathway is regulated within the claudin group of proteins, whereas the permeability of the leak pathway can be regulated by ZO-1, occludin, and myosin light chain kinase (MLCK) [11,12,13]. The tight junctions are lost at sites where epithelial damage, caused by apoptosis, has occurred and the contents of the lumen penetrate the intestinal barrier via the “unrestricted” pathway. As the name suggests, it is a pathway with high capacity and low selectivity, which may explain the development of disease as a result of epithelial damage. This, in the case of erosions or ulcers, is how bacteria gain access to the mucosa [4,14].

The production of anti-inflammatory cytokines can affect the conformation of the junctions between enterocytes, thereby alternating intestinal permeability [15]. For example, pathogen-induced release of interferon γ (INF-γ) increases intestinal permeability through redistribution of TJs proteins and rearrangement of the actin cytoskeleton. Reorganization of TJs triggers the tumor necrosis factor α (TNF-α) to affect intestinal permeability by inducing endothelial cell apoptosis [16]. It has been shown that both claudin-2 (CLDN-2) expression and IL-13 production appear to be higher in ulcerative colitis, in comparison to Crohn’s disease [17,18,19]. In addition, interleukin 6 (IL-6) has also been shown to increase intestinal permeability by stimulating the expression of CLDN-2, which plays a key role in pore formation in the TJs [20]. Disintegration of transepithelial transport pathways may induce further translocation of pathogenic agents, and consequently contribute to the progression of numerous intestinal diseases, such as inflammatory bowel disease (IBD) [4]. Immune mechanisms can directly regulate intestinal permeability. Toll-like receptors (TLRs) are a class of trans-membrane pattern recognition receptors (PRRs) that are important in microbial recognition and play a vital role in controlling the immune response. Type two TLR (TLR2) recognizes conserved structures on both Gram-negative and Gram-positive bacteria. TLR2 is expressed in many cell types in the intestine, including epithelial cells [21]. It has been demonstrated that in-vitro TLR2 stimulation increases transepithelial electrical resistance through the activation of protein kinase C and the translocation of ZO-1 to the tight-junction complex [22].

### 2.3. Mucus

The first line of defense of the intestines is mucus, which is a physical barrier that prevents the adhesion of pathogenic bacteria and other antigens. It consists of water and two types of glycoproteins: secreted mucins (MUC2, MUC5, MUC6) and membrane-bound mucins (MUC1, MUC3, MUC4, MUC13, MUC17) which remain attached to the apical surface and form a glycocalyx together with glycolipids. In the small and large intestine, MUC2 is the most common mucosal protein secreted by goblet cells [23]. MUC2 plays an essential role in epithelial protection, as mice without the *Muc2* gene spontaneously develop severe colitis and inflammation-stimulated colon cancer [24,25]. In the small intestine, mucus comprises a single layer that is rich in antimicrobial substances such as lysozymes, defensins and DMBT1 (Deleted in malignant brain tumors 1 protein) produced by Paneth cells [23,26]. Other mucus proteins secreted by cup cells include the calcium-activated chloride channel regulator-1 (CLCA1), the Fc globulin binding protein (FCGBP), mucus cross-linking proteins, the zymogen 16 protein (ZG16) and a lectin-like protein which binds to Gram-positive bacteria. Secreted mucus mixes with Paneth cell secretions containing antimicrobial peptides, lysozyme, and DMBT1. Immune regulators, such as antimicrobial proteins (AMPs) and IgA molecules, are released in the mucus in a gradient from the epithelium to the lumen, enhancing defense against microorganisms [26]. Mucus does not adhere to the intestinal wall and continually moves in peristaltic waves along intestines, therefore transporting bacteria to the colon [27]. Mucus contained in the large intestine is divided into an inner and an outer layer. The inner layer provides protection from digestive enzymes and, as a result of being impermeable to bacteria, serves as a barrier that separates microorganisms from the epithelium. The outer layer, on the other hand, is not sterile and is colonized by bacteria [28].

### 2.4. Microbiota

Studies on the human digestive microbiome have shown that it comprises over 10^14^ microbial cells belonging to as many as 7000 species. Many of them have not yet been classified because of the complexity of their culturing. The factors that contribute to this issue include the predominance of anaerobic microorganisms, a close relationship with the intestinal mucosa, numerous morphological similarities between the cells and a low uptake of metagenomic methods in routine diagnostics. The diversity and number of microorganisms change with variations in pH and oxygen availability. The intestines—the distal part of the small intestine and the large intestine—show particularly rich colonization [29]. The term *intestinal microbiota* refers to a group of microorganisms that inhabit the lumen of the intestine. It consists of more than 250 different species of bacteria, fungi, viruses and archaea [28,30,31,32,33]. The near-neutral pH level of the ileum environment allows for the survival of numerous relatively anaerobic commensal microorganisms: *Streptococcus* spp., *Lactobacillus* spp. and rod-shaped bacteria from the *Enterobacterales* order, anaerobic members of the *Clostridia* class, as well as yeasts, which are present in quantities of up to 10^7^ CFU/g [34]. Oxygen availability decreases with increasing pH, resulting in peristalsis becoming weaker in the colon and microbial titers upsurging significantly—up to 10^14^ CFU/g of fecal matter. Approximately 70–90% of the species colonizing the large intestine belong to the *Firmicutes* and *Bacteroidetes* families, while the majority of the remaining species belong to the *Proteobacteria* and *Actinobacteria* [28,30,31,32,33]. Nevertheless, their relative abundance differs from site to site and is highly variable between individuals [35]. Bacterial products, such as bacterial toxins, secondary bile acids and short-chain fatty acids produced by fermentation, protect the host against pathogens and fortify the barrier and its functions [34,36,37,38].

The role of microbiota in the context of gut tightness is significant, as it affects the barrier, and elements of the barrier impact the microbiota [27,39]. Studies on germ-free animals show a correlation between a lack of bacterial stimulation in these animals and the thickness of their mucus layers, which is greatly reduced. This suggests that the presence of microflora is essential for the proper functioning of the intestinal barrier [40,41,42,43]. Thinner mucus layers enable penetration by bacteria, which can initiate inflammation and inflammatory diseases, such as colitis. The products of commensal bacteria, such as lipopolysaccharide (LPS) and peptidoglycan, can influence the restoration of the mucus layer [41,43,44]. There is an important interrelation between commensal bacteria and mucus layers which contributes to maintaining intestinal homeostasis, provided that they interact in a balanced manner [42].

Intestinal cells secrete antimicrobial proteins (AMPs) that can remove pathogens and promote colonization by commensal bacteria. Also, the production of some AMPs is regulated by the microbiota and/or its products. Studies on *A. muciniphilia* indicate that commensals regulate the production of the RegIIIγ lectin, which exhibits bactericidal activity against Gram-positive bacteria. Expression of this peptide not only promotes bacterial survival through reduced competition for resources, but also inhibits the growth of pathological strains [45]. Administration of prebiotics or an increase in the number of *Lactobacilli* and *Bifidobacteria* probiotics have shown the capacity to restore RegIIIγ and control bacterial overgrowth [46]. Furthermore, RegIIIγ is an AMP that is essential for the separation of commensal bacteria from the intestinal epithelium [47]. Another example of an AMP with secretion affected by bacteria is the Ang4 protein. A study on Paneth cells in mice showed that the their production was induced by the dominant gut microflora, *Bacteroides thetaiotaomicron* [48]. This shows that the antimicrobial activity of Ang4 against microorganisms in the intestinal lumen is related to the presence of commensal species. Bacteria have also been shown to interact with an intestinal alkaline phosphatase (IAP) protein that is produced mainly by intestinal epithelial cells [49,50]. In comparison with a control wild-type mouse, reduced microbiota and altered bacterial composition were observed in IAP-deficient mice, specifically highlighting a decline in the abundance of *Lactobacillaceae* [51,52]. Due to possessing the ability to deactivate LPS in vivo, IAP plays a vital role in preventing the translocation of pro-inflammatory LPS [53,54]. Increased IAP activity can selectively increase the number of LPS-inhibiting bacteria, such as *Bifidobacterium*, while decreasing the abundance of LPS-producing bacteria, such as *E. coli* [55]. IAP increases the expression of TJs proteins (ZO-1, ZO-2 and occludin) and, therefore, enhances barrier function [56].

There are several other examples of occurrences that demonstrate the diverse effects of bacteria and their metabolites on the integrity of the intestinal barrier. This is especially true in the case of certain probiotic species, including, but not limited to, *Lactobacillus rhamnosus* [57,58,59], *Streptococcus thermophilus* [60], *Lactobacillus reuteri* [61] and *Bifidobacterium infantis*. Moreover, *Bacterioides thetaiotaomicron* stimulates the expression of the small proline-rich protein 2A, which is responsible for the stabilization of desmosomes in epithelial villi [62]. Different strains of *E. coli* have varied effects on the barrier. Namely, *E. coli* Nissle 1917, a probiotic strain, stimulates TJs ZO-2 protein production [45,62], whereas the C25 *E. coli* strain increases permeability [63].

Large changes in the ratio of commensal to pathogenic strains or the growth of new bacterial groups disrupt intestinal homeostasis and may contribute to the pathogenesis or progression of many human diseases, including IBD, autoimmune diseases and metabolic disorders [63]. The aforementioned examples demonstrate how the composition of the microflora significantly modulates the expression of tight junction proteins, the condition of the mucus and the production of inflammatory cytokines.

## 3. Intestinal Infections and Antibiotic Therapy

Antibiotic therapy is a common treatment for bacterial infections in various systems of the body. Studies show that long-term or improper use of antibiotics may contribute to imbalance in the quantity and quality of the complex microbiota ecosystem [29,34,64,65]. The intestinal absorption of antibiotics depends on several factors, including drug properties, intestinal membrane integrity and transport mechanisms. Antibiotics absorbed in the lumen of the intestine have a weaker effect on microbiota. Kim et al. showed that orally administered metronidazole was fully absorbed in the small intestine without affecting the intestinal microbiota [66]. Contrarily, vancomycin, which has a low absorption rate in the gastrointestinal tract, reaches high intestinal levels after oral administration and can significantly affect the reduction of Gram-positive microbiota, thus expanding the niche for *Proteobacteria* [34]. On top of that, Palleja et al. showed that gentamicin, meropenem and vancomycin also increased the frequency of occurrence of *Enterobacteriaceae,* while simultaneously decreasing the number of *Bifidobacterium* species [67]. According to some studies, *Proteobacteria* proliferation creates a state of dysbiosis and contributes to an increased risk of endogenous and exogenous infections [68]. Rifaximin, modulates the secretion of proinflammatory cytokines and, due to low oral bioavailability, has little effect on the composition of gut microbiota [69]. A meta-analysis of 26 randomized controlled trials of adults with small intestinal bacterial overgrowth (SIBO) signified a high rate of SIBO eradication with rifaximin [70]. The accuracy mechanism of action of rifaximin in this case is likely multifactorial and more research is needed; but, in the USA and Canada, rifaximin is indicated in adults with IBS therapy [69]. The *Enterobacteriaceae* family, which is widely distributed in the intestines, also includes pathogenic strains of *Escherichia coli* or *Klebsiella* spp. Furthermore, disturbances in the intestinal microbiota favor *Salmonella enterica* subsp. *enteritidis* and *Clostridioides difficile* infections [66,71]. Nevertheless, it must be noted that overusing antibiotics also leads to the selection of resistant strains and the induction of drug resistance, which is a crucial factor in the context of opportunistic microorganisms that make up the intestinal microbiota [29,72].

Dysbiosis of the microbiome can trigger the release of zonulin, which leads to the contents of the intestinal lumen penetrating the epithelial barrier, therefore releasing proinflammatory cytokines. The presence of cytokines causes a massive influx of food debris and microbial antigens, which leads to the activation of T cells and induces a sustained condition of increased permeability [73]. As a result of microorganism interaction with the intestinal wall, disruption of membrane integrity and profuse watery or even bloodstained diarrhea may occur. This is due to a number of virulence factors that become proliferated by uncontrollably multiplying microorganisms, or to the effect of the toxins they produce. A break in the continuity of the intestinal wall, aside from the adverse effect of toxic waste products or undigested food entering the bloodstream, carries the risk of further pathogenic invasion of the body, leading to severe systemic infections. Infections with etiology of invasive and toxin-producing *Escherichia coli* strains, and proliferation of toxin-producing *Clostridia* representatives, seem to be highly distinctive when regarding intestinal wall integrity disorders.

Enterohemorrhagic *E. coli* (VTEC/STEC/EHEC) are some of the most dangerous pathogens that constitute the etiologic agent of hemorrhagic colitis. Most infections are caused by serotype O157H7 and result from the production of heat-stable cytolysin- a verotoxin belonging to the Shiga toxin family. Upon activation of the MLCK pathway, EHECs affect the redistribution of the occludin and claudin-3 TJs proteins and increase claudin-2 expression without affecting ZO-1 [74]. They also cause a decrease in transepithelial resistance of T84 cells [75]. Hemolytic uremic syndrome can be the result of a further complication of EHEC infection, leading to neurological disorders [76]. Enteropathogenic *E. coli*, although non-invasive, causes defects in the intestinal epithelium by affecting the ultrastructure of its cells [77,78,79]. Thanks to intimin, they coat the intestinal villi and penetrate the cells. EPECs induce MLCK phosphorylation and affect intestinal barrier permeability [80,81]. Numerous secreted proteins, such as EspF, EspG, Map and Tir, cause changes in intestinal epithelial TJs proteins (occludin, claudins, ZO-1/2). In addition, EPEC induces contraction of the actin/myosin ring [82,83,84]. The virulence factors of toxigenic *E. coli* strains (ETEC) responsible for the development of secretory diarrhea are mainly thermolabile enterotoxins. They—LT-1 and LT-2—comprise the A-3B toxin, which is antigenically similar to the *Vibrio cholerae* toxin and to the thermostable STb toxin, which is mainly associated with strains infecting pigs, but also found in ETEC isolated from humans [85]. Through endocytosis, the STb toxin enters the epithelial cells, where it interacts with TJs proteins, namely occludin, claudin-1 and ZO-1 [86]. STb induces nonspecific pore formation in brush border vesicles of the jejunum, and its presence in the cell leads to an increase in Ca^2+^ [85]. Ca^2+^ influx activates calmodulin-dependent protein kinase II, which opens the intestinal ion channel and can activate the kinase C protein and, consequently, lead to CFTR activation [87]. Enteroaggregative *E. coli* (EAEC) strains stimulate the intestine mainly through the adhering aggregates of cell stacks, as well as the production of the EAST toxin, which is similar to the thermostable ST toxin of ETEC strains. The course of the effect on occludin, claudin-1 and ZO-1 involves non-specific interaction of Aggregation Adherence Fimbriae II (AAF II) and its nature is not well comprehended [88]. In 2011 Germany registered an occurrence of the hybrid strain O104:H4, containing the Shiga toxin from the EHEC strain, which caused more than 4300 cases of diarrhea, 50 of which were fatal [89]. The study by Ellis et al. showed that ST40 serotype isolates stimulated IL-8 secretion at higher levels than that of ST31 EAEC strains [90]. Invasive *E. coli* strains penetrate the intestinal epithelium and multiply intracellularly. AIECs, which have strong adhesive abilities (type I fimbriae), stimulate the secretion of inflammatory mediators and are one of the causes of inflammation of the small intestine during dysbiosis associated with Crohn’s disease. They belong mainly to serogroups O6 and O22 [91]. EIECs, although they do not produce toxins, are phylogenetically similar to *Shigella* and distinctively colonize the large intestine where the zonulin system does not function [92,93].

*Clostridioides difficile*, a Gram-positive anaerobic spore-forming bacillus, may be a component of the gut microbiome, but more importantly it constitutes the etiologic agent of pseudomembranous colitis and post-antibiotic diarrhea [29,94]. *C. difficile* toxins are glucosyltransferases that inactivate the Rho family of GTPases [95]. Enterotoxin A (encoded by the *TcdA* gene) exhibits chemotactic activity against multinucleated neutrophils, which leads to infiltration of the ileal wall by leukocytes, release of cytokines, and formation of hemorrhagic necrosis. An increase in inflammatory mediators causes fluid hypertension and watery-bloodstained diarrhea. Cytotoxin B (encoded by the *TcdB* gene) damages the intestinal epithelial cytoskeleton by monoglycosylating proteins involved in actin filament polymerization. Nusrat et al. showed that an increase in paracellular permeability is associated with disorganization of the apical and basal F-actin, accompanied by the dissociation of occludin, ZO-1 and ZO-2 from the lateral TJs membrane, without affecting E-cadherin. In addition, they observed a reduced association of actin with the ZO-1 protein of the cytoplasmic plaque of TJs [94]. It is crucial to note that some *C. difficile* strains produce a cytolethal pervasive toxin (CDT) with an AB2 structure, the role of which in the pathogenesis of CDI (*Clostridioides difficile* infection) remains unidentified and requires further investigation. On the other hand, *Clostridium perfringens*, although mainly associated with gas gangrene, can be an equally dangerous pathogen of post-antibiotic diarrhea. All strains produce phospholipase C, a cytolysin that leads to the degradation of endothelial cells, among others. However, the heat-shock enterotoxin type A, released from cells during spore formation in the ileum and colon by *C. perfringens*, is responsible for gastrointestinal infections, and acts as a superantigen. By stimulating the secretion of cytokines, it damages the intestinal epithelium and increases its permeability [96,97]. The CPE toxin binds to claudin-4 and leads to its destruction [98]. The cytotoxin β2 has a precise affinity for the ileal epithelium and may presumably exacerbate symptoms of prolonged post-antibiotic diarrhea. CPB2 has been shown to damage epithelial cells by interacting with enterotoxins from other bacteria and increasing their uptake [99].

The importance of the intestinal barrier is worth mentioning in the context of the ongoing global pandemic caused by SARS-CoV-2 (COVID-19). An increasing number of research papers show how this virus’ infections establish themselves in the digestive system and how these affect the maintenance of the gastrointestinal tract [100,101]. Assimakopoulos et al. showed that SARS-CoV-2 pneumonia is associated with an increase in the concentration of endotoxins and ZO-1 in serum, which indicates a dysfunction of the intestinal barrier. Additionally, a positive correlation was found between the level of endotoxin and the concentration of CRP and ferritin [102]. Moreover, Prasad et al. presented a relationship between COVID-19 and disturbances in the microbiota, and the tightness of the intestinal barrier. Components of abnormal microbiota in blood were found in almost 65% of patients. The predominance of Gram-negative microorganisms was noted in the study, which also accounted for the increased serum LPS levels found, in comparison with a control group of healthy individuals [103].

The main receptor for the SARS-CoV-2 virus is an angiotensin that converts enzyme 2 (ACE2), which also plays an important role in regulating intestinal amino acid transport, expression of antimicrobial peptides and in preventing intestinal dysbiosis [104]. ACE2 may also affect the intestinal barrier by modifying calcium flow [105]. It has also been speculated that the SARS-CoV-2 virus, itself, can increase intestinal permeability by damaging the epithelial barrier and enterocytes [106].

There are studies in which an intestinal infection model on a chip had been created in order to simulate the colorectal characteristics upon subjection to SARS-CoV-2 infection. Inoculation of the virus induced disturbances in the integrity of intestinal cells and the secretion of mucus and caused morphological damage to the vascular endothelium. The model demonstrated the induction of the following cytokines: TNF, interleukins, chemokines and colony stimulating factors, both in intestinal and endothelial cells [107]. A crucial feature of the study was that the concentration of some cytokines, such as TNF, IL-6, CXCL10, CCL5 and CSF3, was significantly elevated, which corresponds to the clinical results of patients with severe COVID-19 [108,109].

Cohort studies on 100 patients with a confirmed SARS-CoV-2 infection examined the composition of the microbiome in stool, with the concentration of inflammatory markers in the plasma. The results presented significantly altered compositions of the gut microbiome. The virus infection made itself conspicuous through the removal of bacteria with immunomodulatory potential, such as *Faecalibacterium prausnitzii*, *Eubacterium rectale* and some species of *Bifidobacterium*. These changes prevailed for one month after the onset of the infection. The correlation of these changes was presented alongside an increase in the concentration of inflammatory markers such as C-reactive protein, lactate dehydrogenase, aspartate aminotransferase and gamma-glutamyl transferase [110]. Therefore, some authors believe that probiotic therapy, intestinal flora transplantation or probiotic herbal medicine can support COVID-19 therapy and prevent secondary bacterial infections [100,104,111].

## 4. Zonulin

A breakthrough period in understanding the role of intestinal permeability in health and disease was marked by the discovery of zonulin, which is the first human protein to exhibit the regulatory activity of TJs junctions. Presumably, zonulin activates the epidermal growth factor receptor (EGFR) through the proteinase-activated receptor 2 (PAR 2), which leads to phosphorylation of TJs proteins and rearrangement of actin filaments, followed by repression of TJs proteins, which consequently relaxes increasing intestinal permeability [112]. Research programs revealed that zonulin impacts the interactions between bacteria and the host. The increased secretion of this protein has been detected after exposure to pathogenic, as well as non-pathogenic, strains of bacteria [113]. In a study of small intestinal permeability in different species of animals exposed to various bacterial species, a coincidental increase in paracellular intestinal permeability was noted, with disconnection of the ZO-1 protein from TJs [114]. It is assumed that activation of the zonulin pathway may be a defense mechanism that prevents pathogenic bacteria from adhering to, and colonizing, the small intestine. In this case, modulation of gut permeability by activating the zonulin pathway may be an adjunct to the body’s nonspecific response to maintain gut homeostasis. For this reason, zonulin levels cannot always be translated into a pathological clinical state. Another stimulus that is proven to promote zonulin secretion is gluten [115]. Gliadin, which is present in gluten, triggers a zonulin release response by the CXCR3 receptor, activated by coupling with MyD88, with a subsequent increase in intestinal permeability [116,117], suggesting that gluten is misinterpreted by the zonulin pathway as a potentially harmful component of the microflora.

Strategies to modify intestinal barrier function through negative regulation of the zonulin pathway suggest a potential therapeutic application for the treatment of celiac disease. The zonulin inhibitor larazotide acetate presents positive results in the treatment of celiac disease, but still requires extensive studies in a large clinical group [118]. In addition to conventional treatments, several nutritional compounds, including Colostrum bovinum [119], apple pectin [120] and vitamins A and D [121] modulate the epithelial barrier by lowering serum zonulin levels.

Studies in transgenic mice with constitutive activation of myosin light-chain kinase and intracellular mediator of TJs breakdown showed increased intestinal permeability but no signs of disease [122]. Similarly, mice with no JAM-A, which is a key structural component of TJs or muscle myosin IIA heavy chain (NM IIA), exhibited increased intestinal permeability with preservation of the epithelial structure and only mild colitis [123,124]. Finally, transgenic mice constitutively producing high levels of zonulin displayed increased intestinal permeability, but no pathological changes. Furthermore, JAM-A −/− and NM IIA −/− transgenic mice all showed increased susceptibility to chemically induced colitis [73,123,125]. The collective data suggest that intestinal permeability may contribute to disease, provided that additional genetic or microbial factors coexist. In addition to celiac disease, increased intestinal permeability has been reported in the case of other autoimmune diseases, including type 1 diabetes [125], lupus erythematosus [126] and ankylosing spondylitis [127], further highlighting the importance of the paracellular pathway in the pathogenesis of autoimmune diseases. Studies indicating that zonulin is overexpressed during the acute phase of certain chronic inflammatory diseases (CIDs), and that blocking its production prevents the onset of an autoimmune response, suggest that zonulin contributes to the pathogenesis of these conditions, emphasizing previously non-described paradigms in the pathomechanism and treatment options for immune-mediated diseases. Furthermore, it may be inferred that antigen presentation in human macrophages is regulated by zonulin which is associated with altered cytokine profiles and a shift in immune tolerance to autoimmunity [128]. Negative regulation of the zonulin pathway presents a focal point for potential therapeutic measures that target the treatment of chronic autoimmune diseases.

### 4.1. Zonulin and Bacterial Infections

The main catalysts for zonulin release that have been identified to date are bacteria and gliadin, while many enteric pathogens are capable of producing enterotoxins that affect the host’s tight intestinal junctions [73]. El Asmar et al. showed that microorganisms without the zot gene and not secreting Zot, including commensal *Eschericha coli*, laboratory *E. coli*, virulent *E. coli*, and *Salmonella typhimurium,* cause zonulin release from mammalian intestinal wall cells [113]. Once zonulin had been released, the intestines of the test animals displayed increased permeability and reorganization of the tight junction protein complex in the form of ZO-1 detachment. On top of that, Li et al. also found that exposure of Caco-2 cells to *Pseudomonas fluorescens* increased the expression of zonulin [129].

Intestinal barrier dysfunction is a strong determinant of pathogenesis and progression of sepsis. In an experimental model, Yoseph et al. demonstrated that the expression of tight junction proteins is altered during sepsis [130]. In contrast, Klaus et al. found that serum zonulin levels were elevated in patients with sepsis [131]. It has been hypothesized that zonulin may be a key contributor to postoperative sepsis. A study by Liu et al. showed that the use of probiotic therapy in postoperative management reduces the risk of sepsis and correlates with a reduction in serum zonulin levels. Probiotic treatment is also conducive to preservation of the liver barrier, protecting it against metastasis in patients undergoing colon cancer surgery [132]. These data suggest that an increased level of zonulin released from enterocytes leads to migration of bacteria across the epithelium, which may provoke the progression of sepsis. On the other hand, however, studies by El Asmar and Fasano postulate that water secretion into the intestinal lumen, due to zonulin-induced opening of tight junctions, is an independent host defense mechanism. The principle behind this phenomenon is the fact that it leads to flushing the microorganisms out of the intestine, in accordance with the hydrostatic pressure gradient [92,113,133].

### 4.2. Zonulin and Probiotics

Probiotics are live microorganisms that, when administered in appropriate amounts and proportions, provide health-related benefits, of which [134] the most prominent organisms include *Bifidobacterium* and *Lactobacillus* [135]. Probiotics ingested along with prebiotics, which are nutrients for probiotic bacteria, are called synbiotics. Probiotics carry numerous health benefits, which come to fruition by way of increasing the beneficial composition of the intestinal microflora, reducing pathogen adherence, enhancing intestinal epithelial permeability, assisting in regulating the immune response, and ensuring proper metabolic energy levels. Probiotics are generally considered safe and well-tolerated. They have been proven to be advantageous in battling various diseases, such as metabolic disorders, inflammatory bowel disease and colon cancer [136,137,138]. Many studies have shown that probiotics have a beneficial effect on serum zonulin levels, which constitute a measure of intestinal permeability. There are quite a few reports conducted in this department, but their results are still inconclusive. A meta-analysis was generated, selecting nine studies on the effects of probiotic (and synbiotic) intake and serum zonulin levels. The results show that probiotics/synbiotics have a significant effect on reducing serum zonulin compared to placebo groups. It is worth noting that there was considerable heterogeneity in the selected studies. When the analysis was performed separately for the probiotic and the synbiotic, there was a significant reduction observed in subjects who received solely probiotics [139].

Inflammation, obesity and gut microflora in patients with colorectal cancer enable an assessment of the relationship between these aspects. The condition of obesity present in these patients has shown to be associated with changes in the composition and functionality of the gut microbiota. It is characterized by more opportunistic pathogens (such as *Prevotella*, *Fusobacterium nucleatum*, *Enterobacteriaceae* and *Escherichia coli*). Correspondingly, we have found that plasma zonulin levels were significantly higher in obese patients compared to control groups of non-obese intestinal cancer patients, as well as healthy patients. Elevated zonulin levels were attributed to the abundance of *Prevotella* in the gut microbiota of obese patients [140]. *Prevotella* contains enzymes important in mucin degradation that can disrupt the colonic mucosal barrier and impair intestinal barrier function [141]. Although the composition of gut microbiota and its related metabolic functions were correlated with zonulin and calprotectin levels, the study could not distinguish a clear cause for this occurrence, nor its corresponding health repercussions [140].

The disruption of the intestinal barrier integrity has been researched from the standpoint of how it may predispose to metabolic disorders during pregnancy. Changes in serum zonulin levels as a marker of intestinal permeability were recorded, alongside with LPS activity during time of pregnancy. Other interdependencies subjected to research included the effect of ingesting probiotics (*Bifidobacterium animalis* ssp. *lactis* 420 and *Lactobacillus rhamnosus* HN001) and/or supplementation of long-chain polyunsaturated fatty acids (LC-PUFA) on the reduction of serum zonulin levels and LPS activity was checked. In obese pregnant women, as pregnancy progressed, a correlation between the increase in intestinal permeability was demonstrated, which was also reflected in the LPS activity. At the same time, it has not been shown that supplementation with probiotics and/or LC-PUFA significantly influences serum zonulin levels or LPS activity [142].

A group of patients with ulcerative colitis in remission were studied for the effect of multispecies probiotics on intestinal barrier permeability. During the study, there were no significant group- or time-related effects on intestinal permeability, as measured by the 5-sugar absorption test, as well as serum and fecal zonulin concentrations. Similarly, the inflammatory markers C-reactive protein (CRP), calprotectin, and the cytokines IFNc, TNFα, IL-6, and IL-10 were not significantly altered. Urinary sucrose excretion was significantly correlated with serum zonulin and fecal calprotectin. Fecal zonulin was not significantly correlated with any other marker. The authors conclude that serum zonulin may be a more relevant marker of intestinal permeability than fecal zonulin, because of its correlation with other indicators of intestinal permeability. Patients with ulcerative colitis in remission showed no curative effect or change in intestinal permeability when taking probiotics. This should not discourage further studies, as effects may be present during active phase of the disease, or disease exacerbation [143].

Studies on the effects of taking *Bifidobacterium animalis* ssp. *lactis* 420 without and with the presence of fiber showed that blood zonulin levels seemed to consistently decrease throughout the study in these groups, compared with groups taking a placebo or fiber alone. Additionally, a decrease in abdominal body fat and hsCRP was observed [144]. In contrast, there was no significant effect on zonulin levels in migraine patients after taking probiotics [145]. Two-week synbiotic supplementation did not alter intestinal permeability in healthy individuals [146]. Variability in results may also be due to the duration of the trial and the health status of the study group.

## 5. Conclusions

Tight junctions (TJs) between enterocytes—composed of protein complexes—play an important role in controlling intestinal wall permeability. A properly functioning TJs and immune system control the flow of food, as well as bacterial antigens and toxic substances into the intestinal intercellular space (lamina propria). At the same time, the correct qualitative and quantitative composition of the gastrointestinal microbiota plays an important role in maintaining this balance. Dysbiosis caused by the consumption of processed foods, abuse of certain medications (including antibiotics, proton pump inhibitors, non-steroidal anti-inflammatory drugs) or nutritional deficiencies can lead to inflammation, resulting in increased permeability of the intestinal barrier. This results in overloading of the intestinal mucosa with antigens, increased inflammation, impaired absorption and movement of molecules to other distant organs, such as the liver.

The discovery of the protein zonulin, which is the human equivalent of the Zot toxin produced by *Vibrio cholerae*, has made an important contribution to elucidating the link between intestinal barrier permeability disruption and disease pathomechanism. Numerous studies indicate changes in zonulin secretion induced by microorganism presence. It has been observed that a shift in the intestinal microbiota composition balance toward opportunistic microorganisms results in increased serum zonulin levels. Studies on the effect of probiotic microorganisms on zonulin levels have yielded mixed results, but it appears that simultaneous supplementation of Bifidobacterium sp., in combination with a prebiotic, may have a beneficial effect on reducing zonulin levels, in some cases.

The role of zonulin in maintaining the intestinal barrier tightness has been repeatedly demonstrated in studies. It determines the selectivity of the intestinal barrier, which is involved in the control of molecules that penetrate the bloodstream or are retained on the surface of the intestine. Elevated zonulin levels indicate signs of destruction in this barrier, as well as loss of control over the particle passage from the intestinal lumen into the bloodstream. Evaluation of serum zonulin levels appears to be a good marker for assessing intestinal permeability. Numerous publications also demonstrate the effect of the pathogenic microorganism presence and/or dysbiosis on intestinal wall-permeability disorders. Nevertheless, the relationship between microbial interactions and zonulin levels requires further studies to determine the exact relationship between these two factors.

## Data Availability

Data sharing not applicable.
